# Another Use for Padlock Clip

**DOI:** 10.7759/cureus.10656

**Published:** 2020-09-25

**Authors:** Mohamad Awf Mouchli, Vikas Chitnavis

**Affiliations:** 1 Gastroenterology, Cleveland Clinic, Cleveland, USA; 2 Gastroenterology, Carilion Clinic, Roanoke, USA

**Keywords:** polyp, colon polyp, esd, padlock clip, nonlifting

## Abstract

The novel over-the-scope Padlock clip was reported to assist in the management of esophageal fistulas, refractory gastrointestinal (GI) bleeding, and GI defects closure. A little is known about the management of non-lifting colon polyps with fibrosis secondary to prior intervention. Resection could be challenging given prior intervention. The tools used to remove such polyps are different between institutions.

In this study, we describe our experience with Padlock clip in removing non-lifting colon polyp.

## Introduction

A 69-year-old man is referred to our clinic to assist in the management of partially excised large, non-granular, broad-based Tubulovillous adenoma. The polyp was in cecum and measured about 5 cm. The patient underwent a surveillance colonoscopy given the personal history of colon polyps and paternal history of colorectal cancer. The referring physician attempted to remove by standard and Captivator snares using hot snare techniques. The polyp was not completely engaged, and the resected fragments were removed using Roth net.

We attempted to lift the polyp to remove using endoscopic submucosal dissection (ESD), but the scar mark could not be lifted. After marking around the polyp using an electrosurgical knife, endoscopic submucosal dissection was done around the polyp with gradual extension of the circumferential mucosal incision, followed by hot snare-assisted resection of the lesion in a piecemeal fashion. The nonlifting part of the mucosal polyp was then grasped with raptor grasping device and Padlock clip was deployed on the polyp. The remnant polyp was successfully removed with hot snare utilizing "histlock resection device" and the margins were treated with the tip of hot snare. The procedure took about two hours and was not complicated by bleeding or perforation. The final pathology came back as tubular adenoma with low-grade dysplasia. Repeat colonoscopy after three months showed a scar at previous polypectomy site with clean margins and no signs of recurrence.

The Padlock clip (Aponos Medical Co., Kingston, NH) is a new device in the category of over-the-scope clip. It is reported to help in the management of refractory gastrointestinal bleeding [1], fistulas closure [2], and endoscopic full-thickness resection (EFTR) [1].

This device was evaluated in the management of two neuroendocrine tumors in the stomach/duodenum and one sessile rectal polyp. The padlock clip was applied for endoscopic EFTR after invagination of the tissue inside the cap by suction. There was no polyp recurrence within two months and no reported adverse events [1]. EFTR can only be used to resect polyps measuring up to 3 cm [3]. 

To our knowledge, this is the first study describing the use of Padlock clip in the management of nonlifting colon polyp after incomplete resection using the conventional methods. Friedland et al. reported that a combination of piecemeal polypectomy and ablation could help in the management of nonlifting polyps due to previous intervention, but the recurrence rate was 26% [4]. The EndoRotor device (Interscope Medical, Inc., Worcester, MA) was studied in the management of scarred colon polyps and showed a cure rate of 84% [5]. ESD was used in the resection of nonlifting polyps with reduced recurrence rates compared to endoscopic mucosal resection {Saito, 2010 #944}. The use of ESD in resecting nonlifting polyps could be challenging, risky, and time-consuming procedure. Combing the use of padlock clip with ESD could assist in the management of such polyps. 

In conclusion, Padlock clip has a potential role in the management of nonlifting polyps. Further studies are warranted to confirm this role. 

## Technical report

**Figure 1:**
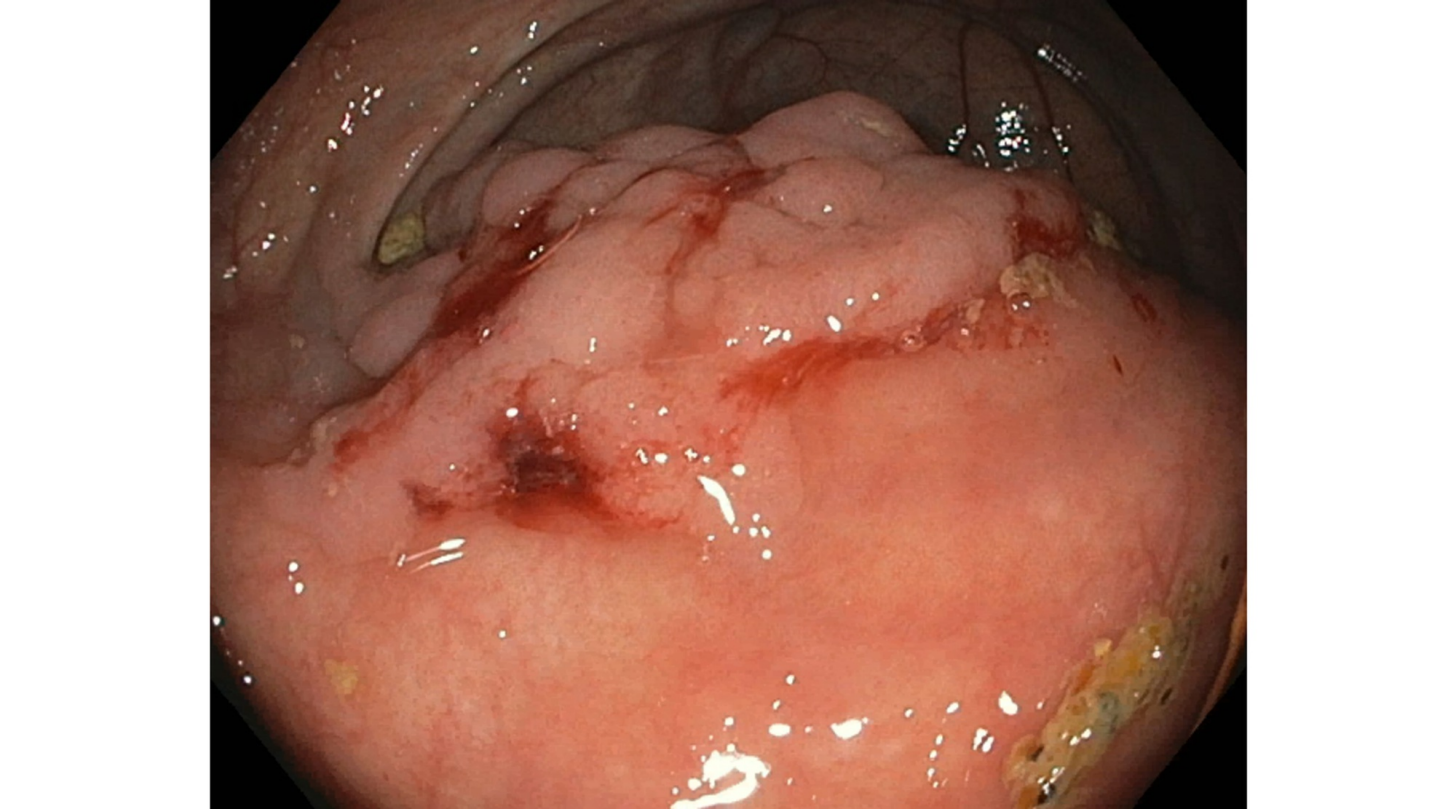
Large cecal polyp measuring 5 cm

**Figure 2:**
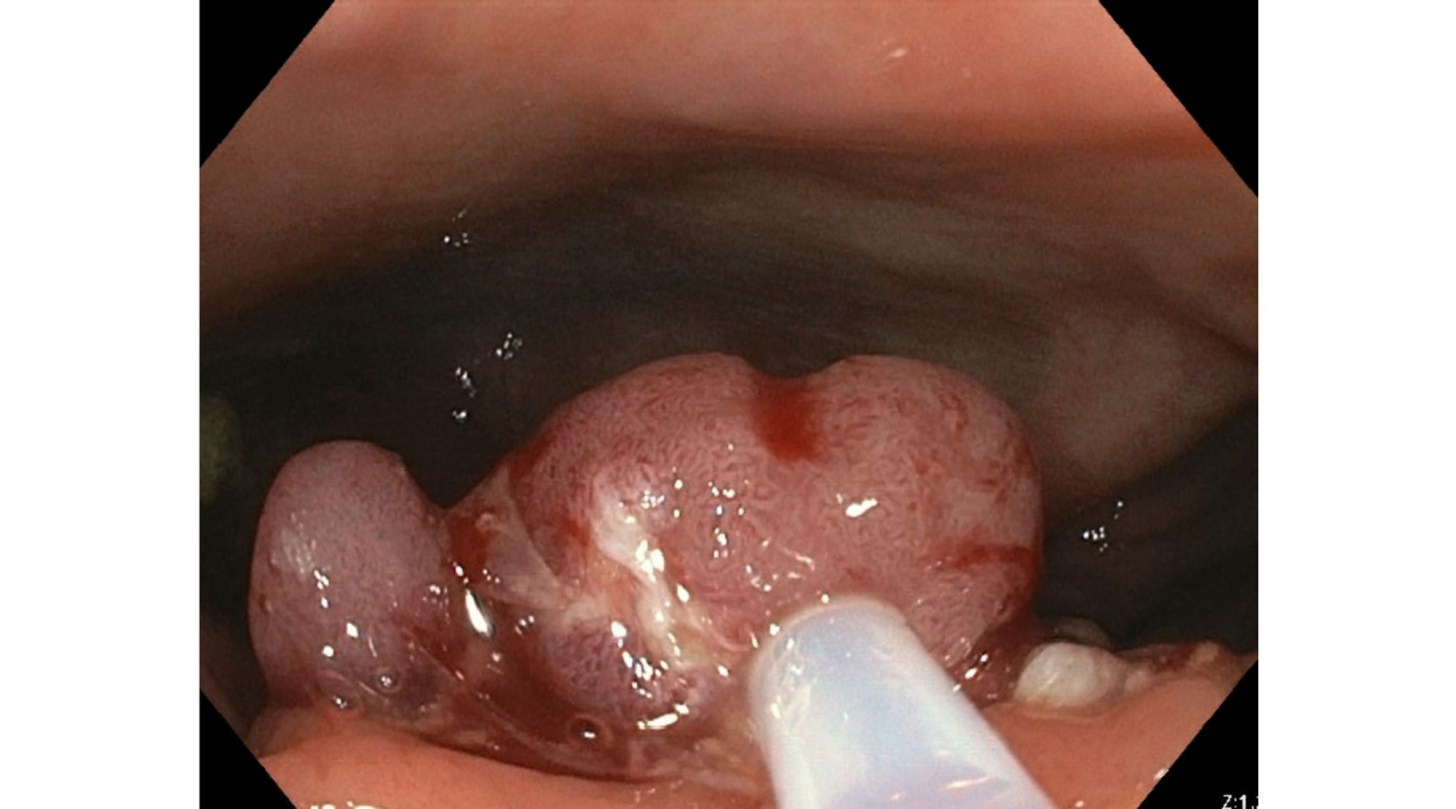
Failed attempt to remove the polyp using standard and Captivator snares

A 69-year-old man is referred to our clinic to assist in the management of partially excised large, non-granular, broad-based Tubulovillous adenoma. The polyp was in cecum and measured about 5 cm (Figure 1). The patient underwent a surveillance colonoscopy given the personal history of colon polyps and paternal history of colorectal cancer. The referring physician attempted to remove by standard and Captivator snares using hot snare techniques (Figure 2). The polyp was not completely engaged, and the resected fragments were removed using Roth net.

**Figure 3:**
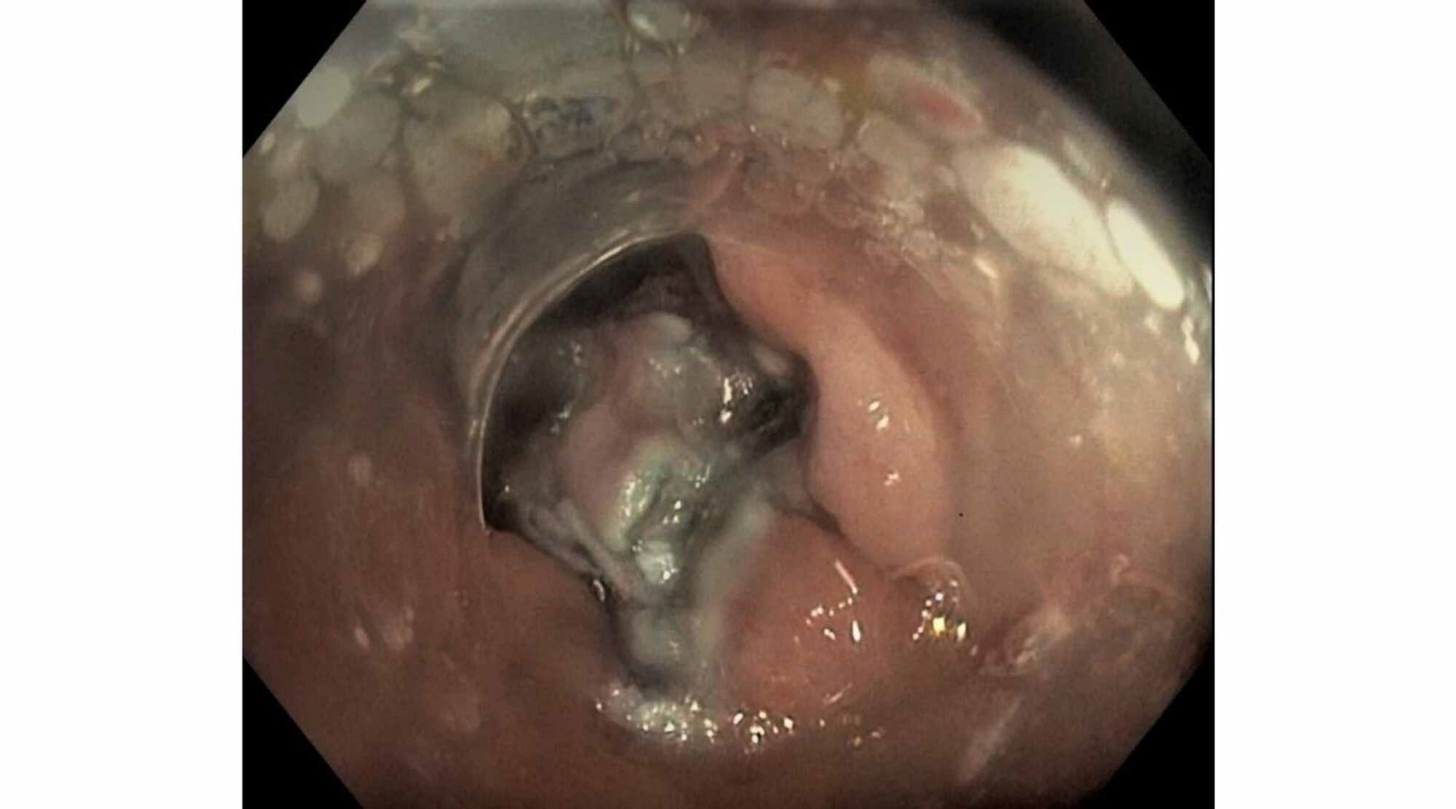
Padlock clip was deployed on the nonlifting part of the polyp

**Figure 4:**
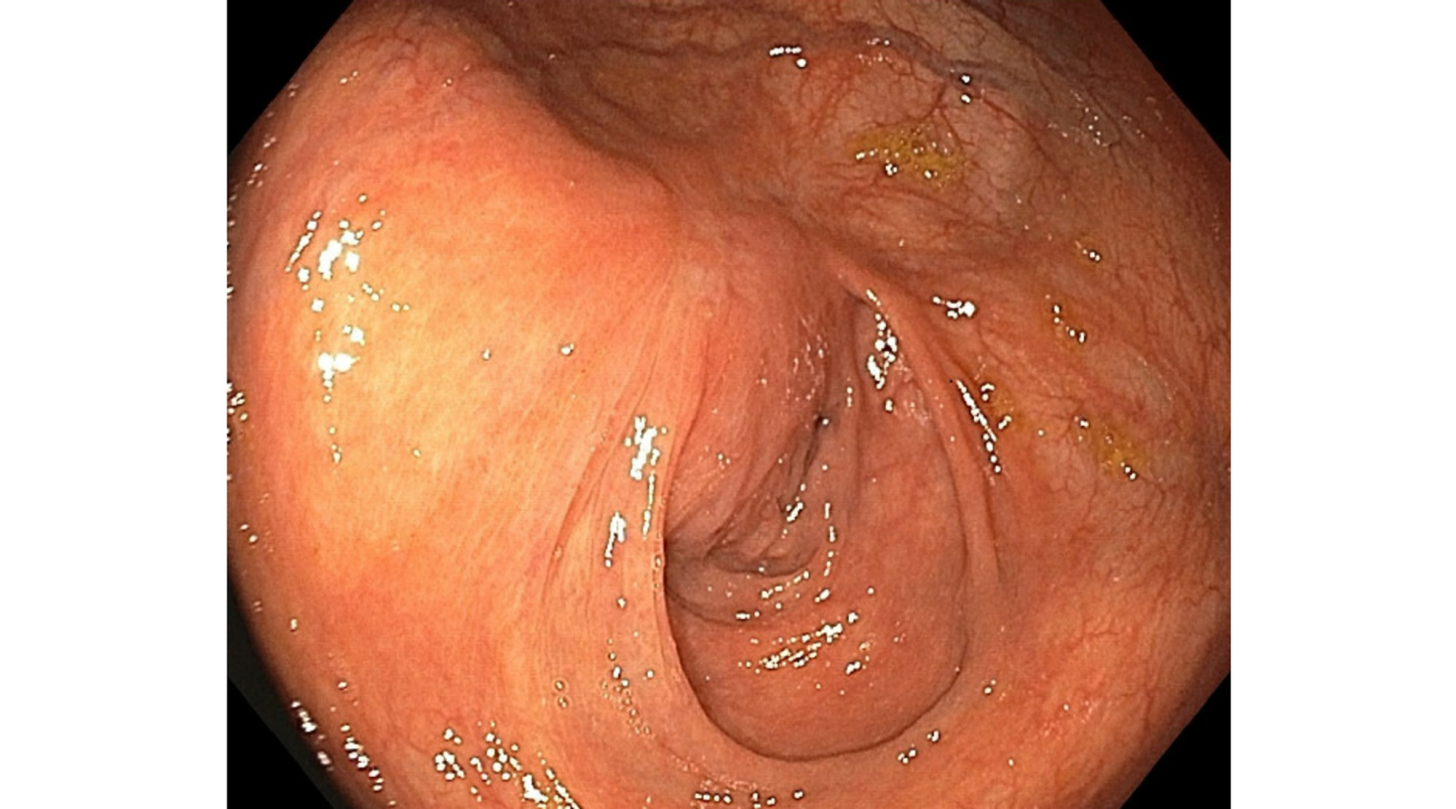
No signs of recurrence on repeat colonoscopy

We attempted to lift the polyp to remove using ESD, but the scar mark could not be lifted. After marking around the polyp using an electrosurgical knife, endoscopic submucosal dissection was done around the polyp with gradual extension of the circumferential mucosal incision, followed by hot snare-assisted resection of the lesion in a piecemeal fashion. The nonlifting part of the mucosal polyp was then grasped with raptor grasping device and Padlock clip was deployed on the polyp (Figure 3). The remnant polyp was successfully removed with hot snare utilizing "histlock resection device" and the margins were treated with the tip of hot snare. Repeat colonoscopy after three months showed a scar at previous polypectomy site with clean margins and no signs of recurrence (Figure 4).

## Discussion

The Padlock clip (Aponos Medical Co., Kingston, NH), is a new device in the category of over the scope clip. It is reported to help in the management of refractory gastrointestinal bleeding [1], fistulas closure [2], and EFTR [1].

This device was evaluated in the management of two neuroendocrine tumors in the stomach/duodenum and one sessile rectal polyp. The Padlock clip was applied for EFTR after invagination of the tissue inside the cap by suction. There was no polyp recurrence within two months and no reported adverse events [1]. EFTR can only be used to resect polyps measuring up to 3 cm [3]. 

To our knowledge, this is the first study describing the use of Padlock clip in the management of nonlifting colon polyp after incomplete resection using the conventional methods. Friedland et al reported that a combination of piecemeal polypectomy and ablation could help in the management of nonlifting polyps due to previous intervention, but the recurrence rate was 26% [4]. The EndoRotor device (Interscope Medical, Inc., Worcester, MA) was studied in the management of scarred colon polyps and showed a cure rate of 84% [5]. ESD was used in the resection of nonlifting polyps with reduced recurrence rates compared to endoscopic mucosal resection [6]. The use of ESD in resecting nonlifting polyps could be challenging, risky, and time-consuming procedure. Combing the use of Padlock clip with ESD could assist in the management of such polyps. 

## Conclusions

Padlock clip has a potential role in the management of nonlifting polyps. Further studies are needed to confirm this role.
